# I believe I can craft! introducing Job Crafting Self-Efficacy Scale (JCSES)

**DOI:** 10.1371/journal.pone.0237250

**Published:** 2020-08-10

**Authors:** Marta Roczniewska, Anna Rogala, Malwina Puchalska-Kaminska, Roman Cieślak, Sylwiusz Retowski

**Affiliations:** 1 Faculty of Psychology, SWPS University of Social Sciences and Humanities, Sopot, Poland; 2 Department of Learning, Informatics, Karolinska Institutet, Procome Research Group, Medical Management Center, Management and Ethics, Stockholm, Sweden; 3 Faculty of Psychology, SWPS University of Social Sciences and Humanities, Warsaw, Poland; 4 Trauma, Health and Hazards Center, University of Colorado, Colorado Springs, Colorado Springs, Colorado, United States of America; Sapienza, University of Rome, ITALY

## Abstract

Job crafting is beneficial for employees and organizations. To better predict these behaviors, we introduce the concept of job crafting self-efficacy (JCSE) and define it as an individual’s beliefs about their capability to modify the demands and resources of their job to better fit their needs. This article describes the development and validation of a scale to measure JCSE. We conducted a qualitative study to design and four quantitative studies to test the psychometric properties of this scale among Polish and American employees in both paper-and-pencil and online versions. Three independent (N_1_ = 364; N_2_ = 432; N_3_ = 403) confirmatory factor analyses demonstrated a good fit to a 3-factor solution comprising JCSE beliefs about increasing (a) structural job resources, (b) social job resources, and (c) challenging job demands. The 9-item JCSE Scale had good internal consistency, high time stability, and good validity. It correlated positively with general self-efficacy. JCSE explained unique variance in job crafting behaviors over and above general self-efficacy, and was more important in predicting job crafting than contextual factors. We demonstrate the role of social cognitions in shaping job redesign behaviors and provide a useful tool to evaluate the effectiveness of interventions dedicated to empowering JCSE.

## Introduction

Job design and redesign are usually seen as top-down processes in which people who manage an organization create jobs and then decide to introduce changes to them [1]. Recently, a new perspective on this phenomenon has been suggested wherein job redesign is understood as a process depending on an individual’s initiative. Unbeknownst to their supervisors, employees may transform the characteristics of their jobs to match them to their needs and preferences. These proactive, bottom-up, and self-initiated actions have been labeled as *job crafting* [1,2]. The current COVID-19 pandemic crisis highlighted the importance of flexibility in how jobs are performed. Thus, employee job crafting may form an especially important organizational asset. Moreover, job crafting has numerous positive consequences for employees and organizations (for a meta-analysis, see: [3]). Therefore, scholars and practitioners are interested in promoting these acts in the workplace (e.g., [4]). When are these behaviors most likely to occur?

Following Social Cognitive Theory [5], we argue that a key step for an employee to engage in job crafting is to build an expectancy that performance of this behavior is within this person’s control. In this project we integrate Social Cognitive Theory and the Job Demands-Resources model [6,7] to introduce the concept of *job crafting self-efficacy* (JCSE). We define JCSE as an individual’s beliefs about his or her own capability to modify demands and resources present at their job to better fit their needs and preferences. We argue that the development of this construct is warranted because scholars and practitioners are interested in finding valid, precise, and useful predictors of job crafting to strengthen these behaviors in the workplace. While the literature points to a list of contextual and individual antecedents to job crafting [3], not all of them are malleable (e.g., personality traits) or easily changed by the organizations (e.g., autonomy levels). This gap does not allow utilizing these factors as enhancers in introducing job crafting to the organizations. Thus, a factor susceptible to interventions and change is practical. In the meta-analysis, general self-efficacy has been positively linked with job crafting behaviors [3]; however, its predictive power was low to medium, and differed for distinct crafting behaviors. We propose that being a context-specific belief, JCSE predicts job-crafting behaviors more precisely than general measures of self-efficacy. By developing a more accurate predictor, we help to explain when job crafting occurs, which is relevant for both research and practice.

We contribute to research and practice in three distinct ways. First, we introduce a malleable, context-specific construct to explain when employees engage in job crafting acts. By integrating Social Cognitive Theory and the Job Demands-Resources model, we explicate how such beliefs are formed and affect behaviors. This knowledge is vital for organizational practice, because it guides development of practices and interventions that may help with introducing job crafting in the workplace. Next, we investigate the interplay between contextual (i.e., perceived opportunities to craft) and individual (i.e., JCSE) factors predicting job self-efficacy to examine their relative importance. An answer to this question may help better guide organizational actions aimed at supporting proactivity in the workplace. Finally, we provide an instrument to measure JCSE beliefs, which was validated across different formats and two languages. This scale may be used to evaluate the effectiveness of interventions dedicated to empower JCSE.

### Job crafting

The term *job crafting* was first coined by Wrzesniewski and Dutton [2] who described the bottom-up process of job re-design, wherein employees proactively introduce changes to certain aspects of their jobs. These authors proposed that individuals craft their jobs by changing their understanding of the role they perform (*cognitive crafting*), by altering the amount or type of tasks they pursue at work (*task crafting*), or by modifying their interactions with other people at work (*relational crafting*). The purpose of these behaviors is to increase the meaning of work and to transform the jobs people have into ones they want to perform [8].

Tims and Bakker [1] proposed an alternative to Wrzesniewski and Dutton’s conceptualization by framing job crafting within the Job Demands-Resources model (JD-R; [9,10]). These authors defined job crafting as a bottom-up process of shaping one’s job characteristics, that is, demands and resources [1]. *Job demands* are those aspects of the job that require physical or psychological effort, and are therefore associated with physiological and psychological costs [7]. Some of the demands, like time pressure, are of a *challenging* character, that is, although they are appraised as stressful, they provide the potential for growth and may have a positive effect on an individual [11]. Other types of job demands, like role conflict, serve as a *hindrance* to effective goal pursuit, and therefore negatively influence an individual [11]. *Job resources* represent those aspects of the job that help buffer the potentially negative impact of job demands on individuals. They support goal pursuit and stimulate personal growth [7]. Examples include work autonomy, feedback, and being provided with learning opportunities. JD-R theory poses that interactions between the levels of job demands and job resources determine employee well-being [7]. Indeed, research demonstrates that high hindrance job demands combined with low amounts of job resources result in exhaustion, whereas high levels of both challenge demands and job resources create employee engagement [7].

Given the assumptions of the JD-R theory, Tims and Bakker [1] argue that employees craft their jobs to achieve higher well-being by optimizing the levels of demands and job resources in their jobs. These authors proposed four crafting strategies. The first two relate to employees increasing their job resources. Individuals can seek more structural job resources, for example, by creating opportunities for development at work or expanding their levels of job autonomy. Employees may also seek more social job resources. For instance, they can look for help or advice from their colleagues to better deal with the demands of their job. Individuals can also optimize the levels of their job demands. They do so by increasing challenging job demands (e.g., introducing new projects in the company), and by decreasing hindering job demands (e.g., reducing workload).

Individuals benefit from job crafting. Those who more frequently initiate changes in their jobs experience higher job satisfaction and work engagement [12], stronger person-job fit [13], increased work meaning [14], and better health [15]. Although the primary aims of job crafting are self-serving, organizations benefit from individual job crafting acts as well. Employees who engage in job-crafting are more productive and have more sustainable employment [16]. Namely, job crafting predicts better in-role and extra-role performance, and weaker turnover intentions [3]. In addition, Ghitulescu [17] found that job crafting is linked with reduced absenteeism.

Given the multiple positive consequences of job crafting, it is important to understand how to encourage it in the workplace. Although job crafting concerns employees' self-initiated actions, these bottom-up re-design behaviors can be supported through organizational interventions (e.g., [15]). Employee intention to engage in these acts combined with managerial encouragement or workplace social norms may not be enough to pursue job crafting. We argue that yet another element has to be added to that equation: an expectancy that performance of the behavior is within a person’s control, that is, they have the capability to implement it successfully. This element is called self-efficacy. Self-efficacy represents people’s beliefs in their capabilities to exert control over the environment and execute actions necessary to deal with future situations [18]. Vough and Parker [19] argue that employees who feel self-efficacious are more likely to act proactively. Indeed, Social Cognitive Theory postulates that people with high self-efficacy levels are more eager to perform challenging or risky tasks [5]. Because job crafting is a demanding activity that requires moving beyond the job descriptions, we believe that the postulates of Social Cognitive Theory apply to this phenomenon. When people with high self-efficacy realize that they are not completely satisfied with their jobs, they perceive that they are in control to change the situation. This realization may lead them to actually alter the characteristics of the job to fit them better [19]. Indeed, a meta-analysis performed by Rudolph and colleagues [3] revealed that general self-efficacy positively correlates with job crafting behaviors.

### Job crafting self-efficacy

Although self-efficacy may be conceptualized and measured in a more global way as the general belief in one’s competence to cope with a broader range of stressful or challenging demands [20], it is usually defined and measured as a domain-specific construct. Social Cognitive Theory argues that self-efficacy measures should be context-specific because self-efficacy itself is a context-specific belief [5,21]. People usually hold distinct efficacy beliefs across disciplines, for example, one can feel very self-efficacious as an academic and, simultaneously, extremely inefficacious as a driver. Indeed, previous studies suggest that applying context-specific self-efficacy measures allows outcomes to be predicted more successfully (e.g., [22]).

To better predict job crafting in the workplace we introduce a new, domain-specific self-efficacy construct: Job Crafting Self-efficacy (JCSE). We theoretically frame our understanding of JCSE within the JD-R resources approach to job crafting. Therefore, we define JCSE as an individual’s beliefs about his or her own capability to modify demands and resources present at their job to adjust them to their needs and preferences. In that, JCSE is different from job crafting itself, because the latter describes employee *acts* of modifications, whereas the former encompasses employee *beliefs* about the ability to perform these modifications. Further, JCSE differs from general self-efficacy, because it is contextualized to focus on one’s beliefs regarding the ability to alter their job characteristics rather than optimistic self-beliefs to cope with a variety of demands in life. Overall, the proposed construct of JCSE combines job crafting and self-efficacy, but is distinct from them.

Because job crafting consists of four conceptually different dimensions in the JD-R model, we propose four corresponding types of JCSE beliefs about one’s ability to: (A) increase structural job resources; (B) increase social job resources; (C) increase challenging job demands; and (D) decrease hindering job demands. We argue that these beliefs form separate dimensions because they describe independent behaviors that employees may engage in to align their jobs with their own preferences. Therefore, we hypothesize that JCSE consists of four sub-dimensions (*Hypothesis 1*). Based on the assumptions of Social Cognitive Theory, we also expect that these specific beliefs predict matching job-crafting behaviors, for example, individuals who feel self-efficacious with respect to increasing challenging job demands are more likely to start new projects or learn about new developments at work and try them out. We hypothesize that there is a positive link between a specific JCSE belief and a corresponding job crafting behavior (*Hypothesis 2*).

Specific JCSE beliefs may derive from more generalized self-efficacy beliefs. Due to the fact that certain levels of hierarchy in self-efficacy have been detected, where general self-efficacy beliefs consist of domain-specific self-efficacy beliefs (e.g., [23]), scholars posit that when individuals encounter recurrent successes in specific contexts, they develop a more generalized self-efficacy. Then, this generalized belief may form the basis for an individual’s assessment of prospective efficacy in new situations [24]. Therefore, we expect a positive correlation between general self-efficacy and JCSE (*Hypothesis 3*). However, because JCSE beliefs are context-specific, we argue that they predict job crafting behaviors more accurately than general self-efficacy (*Hypothesis 4*).

Self-efficacy, next to organization-based self-esteem and optimism, has been included as a personal resource in an extension to the JD-R model [25]. Xanthopoulou and colleagues [25] showed that personal resources mediated the relationship between job resources and engagement. This result indicates that the supply of job resources activates employees' self-efficacy, self-esteem, and optimism, and makes them feel more engaged, thus affecting the *motivational process* described by the JD-R model [7]. The study also showed that personal resources mediated the relationship between job resources and exhaustion. Moreover, previous studies have supported the moderator role of personal resources in the relationship between job demands and well-being (e.g., [26]). Thus, personal job resources affect the *health impairment* process described in the JD-R model. Overall, we expect that JCSE is positively related to work engagement (*Hypothesis 5*) and negatively to job burnout (*Hypothesis 6*).

Finally, we examine the interplay between situational conditions and JCSES in shaping job crafting. Thus, we compare the predictive power of JCSE and a newly-developed construct that measures perceived opportunities to craft (POC) in an organization [27]. This latter construct addresses an individual’s perceptions regarding the possibilities his or her job provides to change certain aspects of it. These opportunities seem dependent on organizational- or job-related factors rather than individual self-efficacy beliefs. Because self-efficacy has both direct and indirect (via intentions) links with actual behaviors [28], we expect JCSE to explain job crafting behaviors above and beyond POC (*Hypothesis 7*).

### Overview of scale development procedure

**Fig 1:**
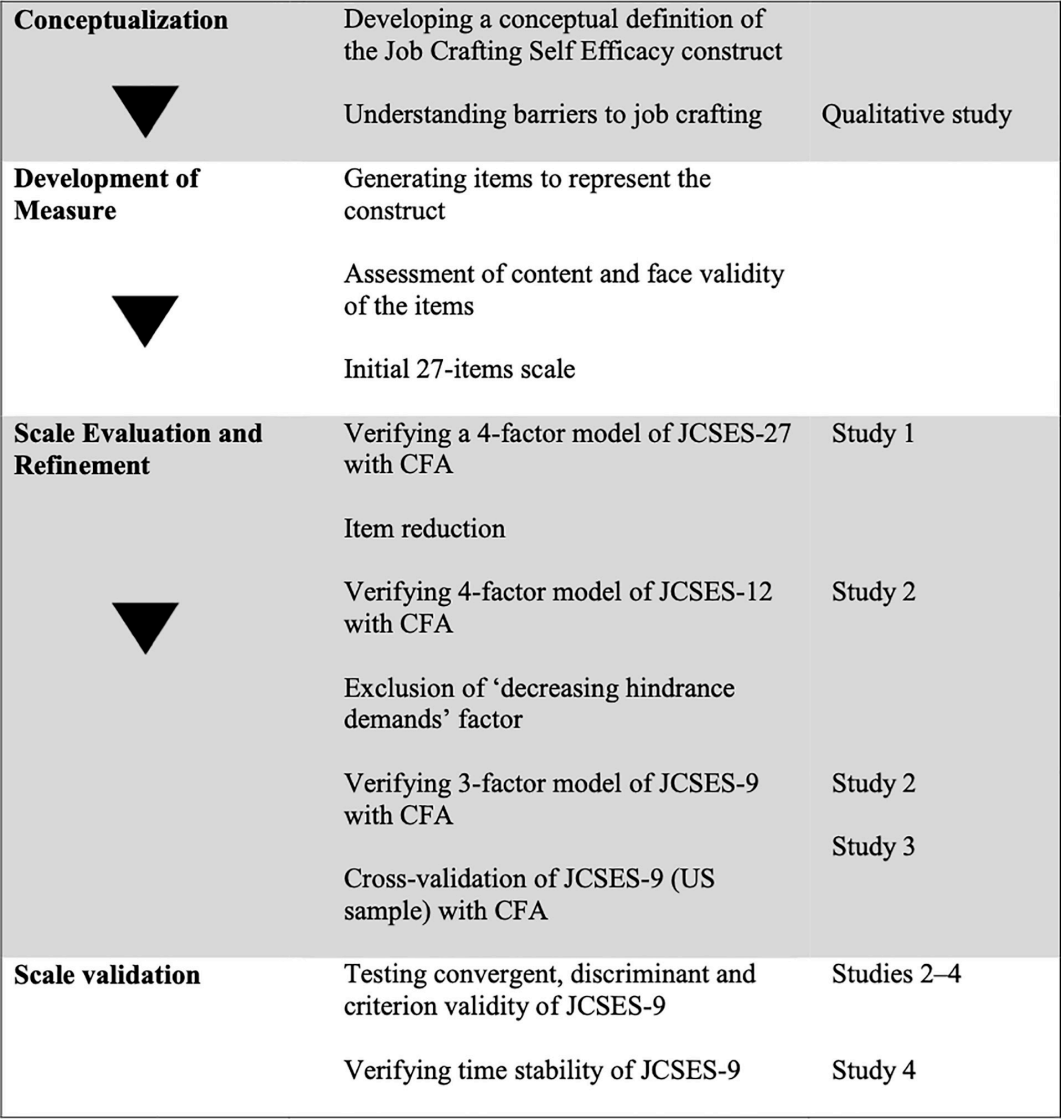
Overview of the Job Crafting Self-Efficacy Scale (JCSES) development procedure.

The aim of our project was to develop a four-dimensional measure for job crafting self-efficacy (JCSE) that uses a limited number of items and can be applied regardless of cultural and occupational context. To achieve this goal, we conducted a series of studies with diverse procedures and samples. As the JCSE Scale (JCSES) was constructed on the basis of the JD-R approach to job crafting [1,29], in line with guidelines [30] we started with conducting a confirmatory factor analysis to verify its four-dimensional factorial structure (*Hypothesis 1*). Then, in order to achieve intended parsimony of the scale, we reduced the number of JCSES items, analyzing its internal and external consistency and judgmental qualities following the guidelines outlined by Stanton, Sinar, Balzer, and Smith [31]. Next, we conducted a series of confirmatory factor analyses among distinct samples and formats, that is, paper-and-pencil (Study 1) and online (Study 2), as well as in an additional cultural context (Study 3). Subsequently, we analyzed JCSES’s convergent and criterion validity (Study 1–4) to test *Hypotheses 2–7* regarding JCSE relations with other constructs. Moreover, we have assessed JCSES’s discriminant validity (Study 2–3). The overview of JCSES development procedure is presented in Fig 1.

## Method

### Constructing of the Job Crafting Self-Efficacy Scale

Three work and organizational psychologists, familiar with job crafting research and Social Cognitive Theory, independently generated an initial pool of items. Following the recommendation for self-efficacy scales [28], self-efficacy items should be represented by confidence-statements with the following semantic structure: "I am confident that I can (perform something) despite (barrier)”. This structure is warranted because human behavior is not only a function of intentions and cognitive control, but also is influenced by the perceived and the actual environment [32]. Thus, introducing barriers provides an opportunity for more realistic assessments [32]. Such structure was used in a variety of scales measuring a wide range of self-efficacy beliefs regarding health behaviors, e.g., physical activity [33], regulation of eating [34] or resisting the urge to use drugs [35]. Hence, following these recommendations, generated items comprised job crafting behaviors along with job crafting barriers. We defined barriers as aspects of the immediate work environment and the self that inhibit the translation of motivation and abilities into job crafting. Typical barriers were selected on a basis of a semi-structured interviews, where participants were asked about constraints to their crafting behaviors (see: S1 Appendix). Examples included lack of energy, hostile relations among employees or a fear of being overwhelmed with additional tasks. We choose the most typical barriers and connected them with specific job crafting behaviors. Specific job-crafting behaviors were chosen on the basis of the JD-R approach to job crafting [29], e.g., asking one’s supervisor for feedback or seeking opportunities for development.

Before we collected data on the JCSES, all authors had discussions about the proposed definition of JCSE and the constructed items. After eliminating clearly redundant items, the initial version of the JCSE Scale consisted of 27 items. Importantly, the items of the JCSE Scale were worded in a simple way (avoiding complex terminology or professional jargon) making the instrument suitable for participants with different levels of education and professional backgrounds. Example items for this initial version of the JCSE Scale include “How certain are you that you would be able to develop your knowledge and abilities despite a heavy workload?” (JCSE in increasing structural resources), “How certain are you that you would be able to ask your supervisor for support, despite concerns about being judged?” (JCSE in increasing social resources), “How certain are you that you would be able to set new job challenges, even though it leads to more responsibilities or a larger workload?” (JCSE in increasing challenges) and “How certain are you that you would be able to reduce how work affects your emotions despite worries about how others will judge you?” (JCSE in decreasing demands). Analogical to other SE scales [21], response categories refer to the certainty of being capable of performing the indicated behaviors in one’s job. We used a 10-point scale, ranging from 1 (*definitely incapable*) to 10 (*definitely capable*). This first version of the scale was assessed to test the factorial validity of the scale and choose items for the final version of the instrument.

### Participants and procedures

A summary of participants’ characteristics across Studies 1–4 is given in Table 1. All studies described in this manuscript were carried out in accordance with the recommendations of Departmental Ethics Committee, who provided specific approval for this research project after the research was reviewed (WKE/S16/V/18). We obtained written informed consent from all subjects in Study 1 and 4, and online consent in Studies 2 and 3, in accordance with the Declaration of Helsinki. All of our studies were based on convenience sampling.

**Table 1:** Summary of Participant characteristics in Study 1–4.

Characteristic	Study 1	Study 2	Study 3	Study 4
Sample size for validity	*N =* 364	*N =* 340	*N* = 381	*N =* 119
Country	Poland	Poland	US	Poland
Gender (percentage of female)	66%	74.8%	48%	58%
Age	*M* = 40.58 (*SD* = 10.32)	*M* = 31.63 (*SD* = 7.56)	*M* = 36.97 (*SD* = 9.52)	*M* = 33.18 (*SD* = 10.33)
Organizational tenure	*M* = 11.79 (*SD* = 9.48)	*M* = 4.73 (*SD* = 4.57)	*M* = 6.65 (*SD* = 5.54)	*M* = 6.16 (*SD* = 8.19)
Education(most common)	Bachelor or master’s degree (58.8%)	Bachelor or master’s degree (81.1%)	Some college or college graduation (65%)	Bachelor or master’s degree (61.3%)
Sector (percentage of private sector organizations)	33.8%	64.3%	70.1%	46%

#### Study 1

The first study had a cross-sectional design. Participants were 364 employees recruited by seven research assistants who distributed paper-and-pencil questionnaires among working adults in Poland. Participants were not remunerated for participation.

#### Study 2

The second study was of a cross-sectional nature as well. Participants were recruited by four research assistants who distributed invitations to the on-line study among Polish working adults. Participants took part in a prize draw to win one out of three vouchers for a bookstore worth about 20 EUR each. Five hundred fifty-four individuals agreed to participate in this research. Missing data were deleted list-wise. Due to participants quitting after completing the first scale in the procedure (i.e., JCSE Scale) and missing data in the next scales, the sample for the subsequent validity analysis was lower (*N* = 340) than for CFA (*N* = 432).

#### Study 3

The third study had a cross-sectional design and was conducted online on the U.S. sample. The scale was translated into English beforehand using a backward translation procedure [36]. Participants were recruited through Mechanical Turk (MTurk)—an online data collection website where individuals register to complete tasks for financial rewards. MTurk was proven to be a reliable source of data collection in several studies (e.g., [37,38]). To be eligible to participate in our study, individuals had to be employed and reside in the United States. Participants were instructed about requirements of the study and its purpose (i.e., research on work attitudes and behaviors). Participants spent approximately 6 minutes on the study. After the completion of the study, each participant was rewarded with $0.75 in compensation. Within the study survey, three validity check items were inserted to control participants’ engagement in the study. There were 418 employees who agreed to participate. Individuals who failed to pass the attention test (*n* = 9) were deleted from the database. Due to missing data and participants quitting after filling in the first measure, that is, JCSES, the final samples for CFA was *N* = 403 and *N* = 381 for the validity analysis.

#### Study 4

The fourth study was conducted by adopting a time-lagged design. The data were gathered using a paper-and-pencil procedure at two measurement points with a six-week time break between Time 1 and Time 2. At Time 1 and 2 participants were asked to mark their questionnaire with a unique code. Additionally, participants were informed at Time 1 that there would be a second measurement point in 6 weeks, and they were kindly asked to attend the study if possible. There were 134 participants at Time 1 and 119 at Time 2. Participants were not remunerated for participation.

### Measures

In each study we measured job crafting self-efficacy as well as actual job crafting behaviors. In Study 2 we additionally tested general self-efficacy and work well-being indicators, i.e., work engagement and job burnout. In Study 3 we also measured general self-efficacy. In Study 4 we assessed perceived opportunity to craft in the organization. All used measures are described below.

#### Job crafting

This construct was assessed in Study 1, 2 and 4 using four sub-dimensions of the Polish version [39,40] of the Job Crafting Scale (JCS) originally developed by Tims, Bakker and Derks [29]. In Study 3 we used the English language version of the JCS. The dimensions are: increasing structural job resources (e.g., “I try to develop my capabilities”), increasing social job resources (e.g., “I ask colleagues for advice”), increasing challenging job demands (e.g., “When there is not much to do at work, I see it as a chance to start new projects”) and decreasing hindrance job demands (e.g. “I make sure that my work is mentally less intense”). Items were rated on a 5-point scale, ranging from 1 (*never*) to 5 (*very often*).

#### General self-efficacy

The variable was measured in Study 2 with the General Self-Efficacy Scale (GSES; [41]) in adaptation by Juczyński [42]. In Study 3 the original English version of GSES was used. The instrument consists of 10 items (e.g., “I can always manage to solve difficult problems if I try hard enough”). Participants respond to these items using a 4–point scale ranging from 1 (*not at all true*) to 4 (*exactly true*).

#### Work engagement

This construct was measured in Study 2 with the short version of the Utrecht Work Engagement Scale (UWES-9; [43]) in adaptation by Cieslak and colleagues [44]. The instrument consists of 9 items (e.g., “At work, I am bursting with energy”, *α* = .90). Participants respond to these items using a 7-point Likert scale ranging from 0 (*never*) to 6 (*always*).

#### Job burnout

This variable was measured in Study 2 with Oldenburg Burnout Inventory (OLBI) [45] in adaptation by Baka and Basińska [46]. The instrument consists of 16 items (e.g., “After my work, I usually feel worn and weary”, *α* = .85). The scale ranged from 1 (*totally disagree*) to 4 (*totally agree*).

#### Perceived opportunity to craft in organization

The variable was measured in Study 4 with Perceived Opportunity to Craft Scale (POCS; [27]) translated into Polish by the authors of this study using a back translation procedure. The scale consists of 5 items (e.g., “At work I have the opportunity to vary the type of task I carry out”). Reliability of the scale was high and stable at Time 1 (*α* = .86) and Time 2 (*α* = .88).

## Results

### Factorial structure of the JCSES

#### Analytical strategy

To verify the factorial structure of JCSES we performed a series of confirmatory factor analyses (CFA) using Mplus 7.0 [47]. Missing data were deleted list-wise. In step 1, we tested for the multivariate normal distribution. Significant coefficients of skewness and kurtosis (*p* < .001) indicate that this assumption cannot be accepted in each reported sample. Consequently, we used the Maximum Likelihood Robust (MLR) estimator instead of Maximum Likelihood (ML) estimator to test the fit. According to a multifaceted approach to an assessment of the model fit, we considered the following fit indicates: Comparative Fit Index (CFI; [48]) and Tucker and Lewis Index (TLI; [49]); Root Mean Square Error of Approximation (RMSEA; [50]) along with 90% confidence interval limits. Additionally, we report traditional chi-square statistics. The model is considered to fit the data when the following values are obtained: RMSEA < .08 [50], and TLI and CFI > .90 [51]. We also tested χ2/df ratio across models, assuming that values below three generally indicate good model fit [52].

#### Confirmatory factor analysis of the initial version of the JCSES

The theoretically supported four-factor model was tested in Study 1 against an alternative one-factor model. This comparison is derived from theory as self-efficacy may also be measured as a more general one-factor construct. The results of the CFA regarding the goodness-of-fit indices of the tested models are presented in Table 2 (see: JCSE-27).

**Table 2:** Confirmatory factor analyses of the JCSES-27 in Study 1 (N = 364), JCSES-12 and JCSES-9 in Study 2 (N = 432), and of the JCSES-9 in Study 3 (N = 414).

Model	*χ2*	*df*	*χ2/df*	RMSEA [90% CI]	CFI	TLI
JCSES-27 (PL)						
One-factor model	1702.90	324	5.26	0.11 [0.10–0.11]	.67	.65
Four-factor model	1167.95	318	3.67	0.09 [0.08–0.09]	.80	.78
JCSES-12 (PL)						
One-factor model	249.75	54	4.63	0.09 [0.08–0.10]	.76	.70
Four-factor model	139.61	48	2.91	0.07 [0.05–0.08]	.89	.84
JCSES-9 (PL)						
One-factor model	140.88	27	5.22	0.10 [0.08–0.12]	.83	.77
Three-factor model	63.15	24	2.63	0.06 [0.04–0.08]	.94	.91
JCSES-9 (US)						
One-factor model	132.89	27	4.92	0.10 [0.08–0.12]	.88	.84
Three-factors model	49.99	24	2.08	0.05 [0.03–0.07]	.97	.96

χ2 = chi-square; *df* = degrees of freedom; *χ^2^/df* = normed chi-square; RMSEA = Root Mean Square Error of Approximation; CFI = Comparative Fit Index; TLI = Tucker-Lewis Index; PL = Polish sample; US = US sample.

The results revealed that the one-factor model of 27-item JCSES fits the data inadequately, with high RMSEA (.108) and the chi-square/*df* ratio above 3 (5.26). The goodness-of-fit indices of the four-factor model are not highly satisfactory; however, they are better than a one-factor model with the results of RMSEA (.09) and the chi-square/df ratio (3.67). The CFI and TLI ratios are not satisfactory in both models: one-factor (CFI = .67; TLI = .65) and four-factor (CFI = .80; TLI = .78). We compared the models using Satorra-Bentler scaled chi-square [53,54]. The results demonstrated that the 4-factor model had a significantly better fit than the 1-factor model, Δχ^2^ = 309.35, Δdf = 6, *p* < .001. Overall, none of the two models had good fit parameters. Thus, we decided to modify the initial version of the JCSES with the aim to reduce the number of items.

#### Scale reduction process

To achieve indented parsimony of the scale we selected three items per subscale. Research suggests that scales consisting of three items may achieve adequate internal consistency reliability [55]. Following the recommendations of Stanton, Sinar, Balzer, and Smith [31] regarding the reduction of the self-reported scales, we first analyzed the internal consistency and then the judgmental qualities of the JCSES. See S1 Table for JCSES-27’s reliability estimates and factor loadings.

Internal consistency refers to the overall degree that items of an instrument are intercorrelated and form a homogenous construct. Since all Cronbach’s alpha reliability estimates were high and range from .80 (JCSE in decreasing hindrance demands) to .90 (JCSE in increasing structural resources), indicating good consistency of the scale [56], we decided to study the factor loadings of items. According to Stanton et al. [31], factor analysis is a popular technique used in the scale reduction as retained items that fail to load strongly on the factor of interest may increase the internal consistency of the scale. Since all of JCSE-27 items had significant and satisfactory loadings (i.e., more than .30; see [31]), we did not have any premises to exclude items upon this criterion.

In the last step, an expert judge panel evaluate the qualities of the subscales such as clarity of expression, the semantic redundancy of an item’s content with other items, and face validity [31]. This criterion of assessment is very important, since respondents might negatively view items that are highly redundant with each other. Following this recommendation, three work and organizational psychology experts analyzed items and chose those that were clear and distinctive from each other. On this basis, we chose 3 items per dimension, resulting in a total of 12 items for future studies.

#### Confirmatory factor analysis of JCSES-12

To verify the proposed four-factor model of the shortened version of JCSES, we performed a confirmatory factor analysis (CFA), using Mplus 7.0 [47] on a sample from Study 2. As Table 2 shows (see: JCSE-12), in line with the results of Study 1, the goodness-of-fit indices suggest that a one-factor model does not fit the data well, with RMSEA higher than .08 (.092) and the chi-square/*df* ratio above 3 (4.63). The four-factor model is satisfactory as for RMSEA (.066) and has an acceptable chi-square/*df* ratio (2.91). However, two out of three items from JCSE in decreasing demands had low factor loadings (below .35) and one of them (“Postpone making tough decisions, despite the pressure from your co-workers”) did not load significantly on the latent factor (*p* = .26) (see Table 3). This result may suggest that these items do not build a coherent latent factor of JCSE in decreasing hindering job demands. Therefore, we decided to reject the four-factor model and in the next step tested an alternative model of JCSE without the dimension of decreasing hindering job demands.

**Table 3:** JCSE scale-12 with factor loadings and Cronbach’s alpha reliability (N = 432).

Job crafting self-efficacy dimension	Item	Factor Loadings	Reliability (Cronbach’s α/ McDonald's ω)
Increasing structural resources	Introduce improvements to the way you perform your job, despite a lack of energy.	.48***	.63/.63
	Seek opportunities for development at work despite minimal support from co-workers and supervisor.	.64***	
	Develop your knowledge and abilities, despite a heavy workload.	.67***	
Increasing social resources	Ask your supervisor for feedback, even though he/she doesn’t give it on his/her own account.	.62***	.65/.69
	Ask your supervisor for support, despite concerns about being judged.	.77***	
	Seek advice from your co-workers, despite their own high workload.	.51***	
Increasing challenging demands	Set new job challenges, even though it leads to more responsibilities or a larger workload.	.76***	.69/.70
	Perform tasks that go beyond your job description, despite concerns of how this may affect your personal life.	.58***	
	Create and initiate new projects, even though the work environment is not supportive.	.64*	
Decreasing hindering demands	Postpone making tough decisions, despite the pressure from your co-workers.	.11	.36/.42
	Limit contact with difficult co-workers or clients, even though the job procedures require	.11	
	Limit the extent to which your job overloads you mentally, despite limited autonomy to decide how to perform your job.	.80***	

*** *p <* .001.

#### Confirmatory factor analysis of JCSES-9

In the next step we verified the hypothesis of the three-dimensional structure of JCSE and contrasted it with the one-factor model. As Table 2 demonstrates (JSES-9 [PL]), the goodness-of-fit indices of the three-factor model regarding CFI and TLI exceed the required .90 (CFI = .94; TLI = .91). Moreover, RMSEA is low (.061) and the chi-square/*df* ratio is lower than 3 (2.63) indicating a good fit. The fit of the three-factor model is significantly better in comparison with the JCSE as a one-factor model Δχ^2^ = 66.63, Δdf = 3, *p* < .001. Additionally, the one-factor model of JCES-9 seems to fit the data rather poorly (RMSEA = .099, the χ2/*df* = 5.22).

We aimed to replicate the factor structure of JSES-9 among a US sample (*N* = 403). As Table 2 shows (JSES-9 [US]), the fit of the three-factor model is significantly better in comparison with the JCSE as a one-factor model Δχ^2^ = 75.33, Δdf = 3, *p* < .001. The goodness-of-fit indices regarding CFI and TLI are higher than 0.90 (CFI = .97; TLI = .96), RMSEA is low (.05) and the chi-square/*df* ratio is lower than 3, indicating a good fit.

To sum up, our series of studies showed that the final version of the JCSE Scale consists of three dimensions—JCSE in: (a) increasing structural resources, (b) increasing social resources, and (c) increasing challenging job demands. To achieve parsimony of the scale, each of JCSE dimensions contains three items. This three-factor structure was confirmed among distinct samples and formats (see S2 Appendix). The final version of the instrument can be found in S3 Appendix. In the next step, we set out to test the convergent and criterion validity of the JCSES.

### Validity of the JCSES

Results regarding convergent and criterion validity are based on correlations and multivariable hierarchical regression analyses. Hypotheses 2–4 regarding criterion validity were verified on samples from cross-sectional online studies: Polish (Study 2) and US (Study 3), and Hypotheses 5–6 about criterion validity with data from Study 2. Hypothesis 7 concerning JCSE’s relative role in comparison with perceived opportunity to craft as predictors of job crafting, and time stability of the scale were verified with data from Study 4 with a time-lagged procedure (Polish sample). Results regarding discriminant validity are based on heterotrait-monotrait (HTMT) ratio of correlations, using data from Study 2 and Study 3. The results of correlations from Studies 2–3 are displayed in Tables 4 and 5.

**Table 4:** Correlations and Cronbach’s alphas (in brackets on the diagonal) among job crafting self-efficacy, job crafting, general self-efficacy, work engagement, and job burnout in Study 2 (N = 340).

	M	SD		1	2	3	4	5	6	7	8	9	10
1. JCSE IStR	6.80	1.69	(.62)										
2. JCSE ISoR	7.09	1.85	.37**		(.65)								
3. JCSE ICD	6.57	1.93	.59**		.49**	(.70)							
4. JC IStR	4.24	.61	.46**		.26**	45**	(.76)						
5. JC ISoR	3.19	.85	.08		.48**	.20**	.30**	(.73)					
6. JC ICD	3.55	.87	.40**		.30**	.56**	.59**	.37**	(.82)				
7. JC DHD	2.98	.73	-.14*		-.05	-.19**	-.07	.03	-0.8	(.68)			
8. GSE	3.17	.42	.35**		.27**	.31**	.37**	.16**	.45**	-.04	(.89)		
9. WE	4.50	1.01	.29**		.23**	.39**	.47**	.38**	.52**	-.23**	.29**	(.90)	
10. JB	2.77	.59	-.30**		-.23*	-.23**	-.37**	-.29**	-.37**	.27**	-.27**	-.66**	(.86)

JCSE = Job crafting self-efficacy; JC = Job crafting; IStR = increasing structural resources; ISoR = increasing social resources; ICD = increasing challenging demands; DHD = decreasing hindering demands; GSE = general self-efficacy; WE = work engagement; JB = job burnout.

*p < .05.

**p < .01.

**Table 5:** Correlations and Cronbach’s alphas (in brackets on the diagonal) among job crafting self-efficacy dimension, job crafting and self-efficacy in Study 3 (N = 381).

	*M*	*SD*	1	2	3	4	5	6	7	8
1. JCSE IStR	7.00	1.63	(.79)							
2. JCSE ISoR	6.78	1.79	.55***	(.74)						
3. JCSE ICD	6.55	1.74	.73***	.51***	(.73)					
4. JC IStR	3.80	.64	.59***	.39***	55***	(.77)				
5. JC ISoR	2.83	.86	.27***	.41***	.33***	.46***	(.84)			
6. JC ICD	3.22	.81	57***	.34***	.60***	.71***	.57***	(.81)		
7. JC DHD	2.87	.81	-.08	-.09	-.08	.06	.21***	.09	(.82)	
8. GSE	3.24	.46	.45***	.35***	.33***	.53***	.15**	.37***	-.12*	(.90)

JCSE = Job crafting self-efficacy; JC = Job crafting; IStR = increasing structural resources; ISoR = increasing social resources; ICD = increasing challenging demands; DHD = decreasing hindering demands; GSE = general self-efficacy.

*p < .05.

**p < .01.

***p < .001.

In line with Hypothesis 2, there are positive and moderate correlations between each dimension of JCSE and its parallel job crafting dimension, that is, increasing structural resources (Study 2: *r* = .46, see Table 4, *p* < .001; Study 3: *r* = .59, *p* < .01, see Table 5), social resources (Study 2: *r* = .48, see Table 4 *p* < .001; Study 3: *r* = .40, *p* < .01, see Table 5), and challenging job demands (Study 2: *r* = .56, *p* < .001, see Table 4; Study 3: *r* = .60, *p* < .01, see Table 5).

Supporting Hypothesis 3, we found significant and positive correlations between general self-efficacy and each dimension of JCSE, i.e., increasing structural resources (Study 2: *r* = .35, *p* < .001, see Table 4; Study 3: *r* = .45, *p* < .001, see Table 5), social resources (Study 2: *r* = .27, *p* < .001, see Table 4; Study 3: *r* = .35, *p* < .001, see Table 5) and challenging job demands (Study 2: *r* = .31, *p* < .001, see Table 4; Study 3: *r* = .33, *p* < .001, see Table 5).

To verify whether each JCSES dimension has a stronger impact on specific job crafting behavior than general self-efficacy (Hypothesis 4), we conducted multivariable hierarchical regression analyses among Polish and US samples. In each model with job crafting behaviors as explained variables, general self-efficacy (GSE) was entered in the first step (Model 1), and in the second step we entered a relevant JCSE dimension (Model 2). The increment in R-square was considered as a measure of the added value of the variable entered in the second step, and thus of its lack of redundancy with respect to what is explained in the first step [57].

Table 6 presents results of regression analyses for job crafting dimensions in studies 2 (Poland) and 3 (US). As Table 6 indicates, adding JCSE in step 2 contributed to additional unique explained variance in each type of job crafting behavior across both samples. This result means that a relevant JCSE belief predicts additional variance in job crafting behaviors over and above GSE. The results among Polish and US samples are comparable.

**Table 6:** Results of the multivariable hierarchical regression analysis in Study 2 (N = 340) and Study 3 (N = 381).

Study 2: Poland							
		JC increasing structural resources		JC increasing social resources		JC increasing challenging demands	
Variable		Model 1 *β*	Model 2 *Β*	Model 1 *β*	Model 2 *β*	Model 1 *β*	Model 2 *β*
General self-efficacy		.37***	.25***	.16**	.03	.45***	.30***
Job crafting self-efficacy (relevant dimension)			.37***		.47***		.47***
*R*^*2*^ (adjusted R)		.14 (.14)***	.26 (.26)***	.03 (.02)**	.23 (.23)***	.20 (.20)***	.40 (.40)***
Δ*R*			.12***		.21***		.20***
Study 3: US							
		JC increasing structural resources		JC increasing social resources		JC increasing challenging demands	
Variable		Model 1 *β*	Model 2 *Β*	Model 1 *β*	Model 2 *β*	Model 1 *β*	Model 2 *β*
General self-efficacy		.53***	.33***	.15**	.01	.37***	.20***
Job crafting self-efficacy (relevant dimension)			.44***		.41***		.54***
*R*^*2*^ (adjusted R)		.28 (.27)***	.43 (.43)***	.02 (.02)**	.17 (.16)***	.14 (.14)***	.40(.40)***
Δ*R*			.15***		.14***		.26***

JC *=* Job Crafting

*p < .05.

**p < .01.

***p < .001.

In both samples, GSE predicted increasing structural resources (Study 2: *β* = .37, *p* < .001; Study 3: *β* = .53, *p* < .001) in Model 1; however, after entering JCSE in increasing structural job resources (Model 2), GSE’s impact decreased (Study 2: *β* = .25, *p* < .001; Study 3: *β* = .33, *p* < .001). JCSE in increasing structural job resources positively predicted increasing structural resources (Study 2: *β* = .37, *p* < .001; Study 3: *β* = .44, *p* < .001), and explained additional variance (Study 2: Δ*R*^*2*^ = .12, *p* < .001; Study 3: Δ*R*^*2*^ = .15, *p* < .001;) over and above GSE. General self-efficacy also predicted increasing social resources (Study 2: *β* = .16, *p* = .003; Study 3: *β* = .15, *p* = .003); however, its impact stopped being significant with job crafting self-efficacy in increasing social resources (Study 2: *β* = .03, *p* = .520; Study 3: *β* = .01, *p* = .833) as a predictor in Model 2. JCSE in increasing social resources was a positive predictor of increasing social resources (Study 2: *β* = .47, *p* < 001; Study 3: *β* = .41, *p* < .001) and explained additional variance in increasing social resources (Study 1: Δ*R*^*2*^ = 21%; Study 2: Δ*R*^*2*^ = 14%). Finally, general self-efficacy was a stronger predictor of increasing challenging demands in Model 1 (Study 2: *β* = .45, *p* < .001; Study 3: *β* = .37, *p* < .001), than in Model 2 (Study 2: *β* = .30, *p* < .001; Study 3: *β* = .20, *p* < .001). When JCSE in increasing challenging demands was entered in Model 2, both variables were a positive predictor of job crafting through challenges, but JCSE was a stronger one (Study 2: *β* = .47, *p* < .001; Study 3: *β* = .54, *p* < .001). JCSE in increasing challenging demands contributed significantly to the prediction of how often participants sought challenges (Study 2: Δ*R* = .20, p < .001 Study 3: Δ*R* = .26, p < .001).

In Hypothesis 5 we predicted that all types of JCSE would be positively related to work engagement (WE). In Study 2 we found positive and low relations between WE and JCSE in increasing both structural (*r* = .27, *p* < .001, see Table 4) and social (*r* = .22, *p* < .001, see Table 4) job resources. The relation between WE and JCSE in increasing challenging job demands was positive and moderate (*r* = .40, *p* < .001, see Table 4).

In line with Hypothesis 6, Study 2 demonstrated that relations between JSCE dimensions and burnout are significant and negative. These relations were similar in strength and low for each JCSE dimension, that is, JCSE in increasing structural resources (*r* = -.30, *p* < .001, see Table 4), JCSE in increasing social resources (*r* = -.23, *p* < .001, see Table 4), and JCSE in increasing challenging demands (*r* = -.25, *p* < .001, see Table 4).

To verify whether JCSE predicts job crafting better than perceived opportunity to craft in an organization (Hypothesis 7), we utilized a time-lagged design (Study 4) with predictors measured at Time 1, and explained variables—at Time 2. We used a multivariable hierarchical regression analysis with POCS measured at Time 1 entered in step 1 (Model 1), and a relevant JCSE dimension measured at Time 1 and added as a second step variable (Model 2). Relevant job crafting behaviors measured at Time 2 acted as an explained variable. Table 7 presents study correlations and Table 8 presents the results of the regression analysis.

**Table 7:** Correlations and Cronbach’s alphas (in brackets on the diagonal) between variables from Time 1 and Time 2 measured in Study 4 (N = 119).

	*M*	*SD*	1	2	3	4	5	6	7	8	9	10	11	12	13	14	15	16
1. JCSE IStR T1	7.04	1.81	(.81)															
2. JCSE ISoR T1	5.75	2.11	.42**	(.69)														
3. JCSE ICD T1	6.16	2.12	.65**	.55**	(.83)													
4. JC IStR T1	4.02	.73	.60**	.24**	.46**	(.80)												
5. JC ISoR T1	2.48	1.15	.26**	.37**	.23*	.30**	(.88)											
6. JC ICD T1	3.28	.86	.61**	.34**	.74**	.55**	.42**	(.85)										
7. JC DHD T1	3.20	.78	.07	-.08	.08	.19*	.21*	.10	(.76)									
8. POC T1	4.31	1.42	.48**	.47**	.58**	.39**	-.02	.43**	.11	(.86)								
9. JCSE IStR T2	6.77	1.77	.77**	.50**	.63**	.41**	.14	.55**	.03	.57**	(.81)							
10. JCSE ISoR T2	5.97	2.21	.46**	.76**	.58**	.18*	.25**	.35**	-.02	.57**	.67**	(.83)						
11. JCSE ICD T2	6.19	1.97	.59**	.59**	.77**	.40**	.21*	.62**	.02	.57**	.73**	.72**	(.82)					
12. JC IStR T2	3.98	.73	.57**	.25**	.49**	.82**	.33**	.57**	.18*	.46**	.51**	.27*	.48**	(.83)				
13. JC ISoR T2	2.46	1.14	.20*	.35**	.21*	.27**	.89**	.38**	.21*	-.02	.11	.25*	.22*	.30**	(.89)			
14. JC ICD T2	3.27	.88	.54**	.36**	.66**	.49**	.45**	.79**	.17	.47**	.54**	.46**	.65**	.57**	.49**	(.87)		
15. JC DHD T2	3.26	.77	.20*	.02	.08	.37**	.32**	.22*	.82**	.07	.09	-.01	.06	.30**	.32**	.18	(.80)	
16. POC T2	4.34	1.39	.45**	.41**	.62**	.36**	.04	.52**	.15	.85**	.55**	.55**	.62**	.46**	.04	.52**	.11	(.88)

JCSE = Job crafting self-efficacy; JC = Job crafting; IStR = increasing structural resources; ISoR = increasing social resources; ICD = increasing challenging demands; DHD = decreasing hindering demands; POC = Perceived opportunity to craft.

*p < .05.

**p < .01.

**Table 8:** Results of the multivariable hierarchical regression analysis in Study 4 (N = 119).

		JC increasing structural resources		JC increasing social resources		JC increasing challenging demands	
Variable		Model 1 *Β*	Model 2 *β*	Model 1 *β*	Model 2 *β*	Model 1 *β*	Model 2 *β*
Perceived opportunity to craft		.46***	.24**	-.02	-.24*	.47***	.13
Job crafting self-efficacy (relevant dimension)			.46***		.46***		.58***
*R*^*2*^ (adjusted R)		.21 (.20) ***	.37 (.36)***	-.00 (-.01)	.16 (.15***	.22 (.21)***	.44(.43)***
Δ*R*			.16***		.16***		.23***

*p < .05.

**p < .01.

***p < .001.

The results suggest that POC has a lower predictive power than JCSE for job crafting behaviors. POC predicted increasing structural resources (*β* = .46, *p* < .001) in Model 1; however, after entering JCSE in increasing structural job resources (Model 2), POC’s impact decreased (*β* = .24, *p* < .001). JCSE in increasing structural job resources positively predicted increasing structural resources (*β* = .46 *p* < .001), and explained additional variance (Δ*R*^*2*^ = .16, *p* < .001) over and above POC. Surprisingly, POC did not predict job crafting in increasing social resources (*β* = -.02, *p* = .84). As expected, JCSE in increasing social job resources positively predicted increasing structural resources (*β* = .46 *p* < .001), Model 2 indicated that when JCSE in increasing social resources entered the model, the link between POC and job crafting in increasing social resources became significantly negative (*β* = -.24 *p* = .016). Finally, POC is a significant predictor of increasing challenging demands in Model 1 (*β* = .47, *p* < .001), but not in Model 2 (*β* = .13, *p =* .13). When JCSE in increasing challenging demands is entered into Model 2, POC’s influence is no longer significant and JCSE’s influence is strong (*β* = .58, *p* < .001). JCSE in increasing challenging demands contributed significantly to the prediction of how often participants sought challenges (Δ*R* = .23, *p* < .001).

Finally, to verify discriminant validity of JCSES we investigated if the JCSE constructs are different from job crafting behavior dimensions, as well as from GSE. For this purpose, we used heterotrait-monotrait (HTMT) ratio method [58] for both Study 2 and 3. The HTMT test ‘requires the calculation of a ratio of the average correlations between constructs to the geometric mean of the average correlations within items of the same constructs’ [59, p. 124]. The 0.85 threshold was used to indicate if the two constructs are distinguishable [58]. The results of both Study 2 and Study 3 suggest that all JCSE dimensions show discriminant validity with respect to dimensions measuring job crafting behaviors, as well as to GSE. All ratios resulting from the HTMT analyses were lower than 0.85 cut-off point, and ranged from 0.12 to 0.78.

### Internal consistency analyses and time stability

Cronbach’s alpha values for all JCSE dimensions in Studies 2–4 ranged from .62 to .83, which is acceptable in preliminary research [60]. What is more, we examined test-rest reliability of Time 1 and Time 2 responses on the JCSE Scale by testing correlations between the two measurement points. As Table 7 demonstrates, the results of the correlation analyses between job crafting self-efficacy dimensions measured both times are similar and high, that is, *r* = .77, *p* < .001, for JCSE increasing structural resources; *r* = .76, *p* < .001, for JCSE increasing social resources; *r* = .77, *p* < .001, for increasing challenging demands.

## Discussion

In this paper we introduced the concept of Job Crafting Self-Efficacy and evaluated the psychometric characteristics of the JCSE Scale. Job crafting self-efficacy is a person’s beliefs about his or her own capability to modify demands and resources present at their job to better fit their needs and preferences. The results of our research suggest that JCSE predicts actual job-crafting behaviors. These findings are consistent with Social Cognitive Theory [5,21] according to which self-efficacy beliefs ‘play a key role in shaping the courses lives take by influencing the types of activities and environments people choose to get into’ [61, p. 10].The key strength of our studies is developing a more accurate predictor of job crafting behaviors, which has both theoretical and practical implications. Below we expand on these contributions.

### The Job Crafting Self-Efficacy Scale

The present series of studies showed that the final version of the JCSES (JCSES-9) consists of three dimensions—JCSE in: (a) increasing structural resources, (b) increasing social resources, and (c) increasing challenging job demands. In the first two studies we tested a four-factor model of JCSE, including the dimension of JCSE in decreasing hindering job demands (JCSES-12). In Study 2, the four-factor model did not fit the data well; therefore, based on the results of confirmatory factor analysis, we proposed an alternative, three-factor model where we dropped “JCSE in decreasing hindering job demands” as a factor. The three-factor model of JCSE is supported not only by statistical means, but is also sound given the results of two recent meta-analyses on job crafting. Rudolph and colleagues [3] found that there is low factor loading and a small amount of variance explained in overall job crafting by the dimension of decreasing hindering job demands. Moreover, there is no link between this latter construct and general self-efficacy [3]. It is therefore possible that decreasing hindering job demands operates differently than other job crafting dimensions. Similarly, Lichtenthaler and Fischbach [62] proposed a distinction between promotion-focused job crafting (increasing resources and challenging demands) and prevention-focused job crafting (decreasing hindering demands). These authors demonstrated that there is a positive relationship between promotion-focused job crafting and health, motivation and performance, while there is a negative relationship between prevention-focused job crafting and the aforementioned variables. It seems, therefore, that promotion-focused job crafting offers a more constructive way of optimizing the fit between one’s job and their needs and preferences than a prevention-focused one. Thus, we suggest that it is possible to differentiate between personal beliefs about one’s abilities to perform promotion- versus prevention-focused job crafting behaviors, and that the JCSES-9 developed here can be used to measure the first group of beliefs. Recently, scholars have introduced the facet of job crafting which relates to *optimizing* (rather than avoiding) job demands [63]. Initial research shows that optimizing demands is positively related to work engagement, which implies that—in contrast to decreasing hindering job demands—it may be a favorable behavior [63]. However, because optimizing job demands was not a part of the initial job crafting model [1], as well as the corresponding scale to measure job crafting behaviors [29], we have not included this in our theorizing. However, we encourage future researchers to develop JCSE to capture self-efficacy in optimization of job demands.

The results of our research support the validity of the JCSE-9. Across Polish (Study 2) and US samples (Study 3) all JCSE dimensions were positively associated with GSE. Moreover, JCSE dimensions were predictive of their parallel job crafting dimensions (Studies 2–4). We also showed that specific JCSE dimensions predicted actual job crafting behaviors more accurately than general self-efficacy beliefs. Further, JCSE-9 dimensions were positively related to work engagement, and negatively—to job burnout. This result supports the positive role of personal job resources in motivational and health impairment processes as described by the JD-R model [6]. What is more, results of discriminant validity analysis showed that JCSE dimensions are different from dimensions measuring job crafting behaviors, as well as from a measure of GSE. Thus, the results suggest that JCSES, self-efficacy scale and job crafting scale do not overlap. Finally, evidence from of our research showed that the distinguished dimensions can be reliably measured with our instrument as JCSES-9 showed good internal consistency estimates (Studies 1–4) and high stability over time (Study 4). Importantly, the results of our studies showed that the three-factor model had significantly better fit than the one-factor model. In this sense, it follows the results for job crafting instruments, where two recent meta-analyses [3,62] revealed that specific job crafting dimensions may have distinct relationships with individual differences, job characteristics, and work outcomes. For example, only increasing structural resources and increasing challenging demands have negative relationships with turnover intentions. On the other hand, increasing social resources and increasing challenging demands are weakly, but positively associated with prevention focus [3]. These and our findings, combined with the results from CFAs, discourage from using a total score when assessing job crafting behaviors. Therefore, we highly recommend using the JCSES-9 subscale scores rather than the total score.

### Theoretical and practical implications

In our series of studies, we introduced the concept of JCSE and consequently contributed to the theory and practice in three distinct ways. First, our research demonstrated that JCSE, as a context-specific belief, is a more accurate predictor of job crafting than general self-efficacy. Thus, we introduced a predictor that is not only more precise, but its advantage (as compared to, for example, personality traits) is the potential for malleability. Self-efficacy is a modifiable belief [5], which may be successfully enhanced using psychological interventions [44,64]. Techniques enhancing self-efficacy that are used in these interventions are usually based on sources of self-efficacy listed by Bandura [18], for example, vicarious or mastery experiences. Therefore, we propose that it may be possible to promote job crafting behaviors through JCSE interventions. For example, during workshops employees may be encouraged to share successful attempts in job crafting and plan how to overcome barriers that may interrupt the job crafting plans that they develop. Moreover, since leaders have significant impact on employees [65], we believe that they can enhance JCSE among their subordinates through acting as role models (i.e., vicarious experience) by demonstrating how they craft their jobs. Moreover, leaders can use verbal persuasion to encourage employees to craft. On a day-to-day basis, they may use feedback and appreciate employees’ crafting attempts. Further experimental investigations are needed to evaluate the effectiveness of this type of interventions.

Second, the evidence from Study 4 also suggests that JCSE is more relevant for job crafting than perceived opportunity to craft in an organization. An implication of this result is the possibility that in case of proactive behaviors, such as job crafting, the predictive power of personal resources may be stronger than factors that relate to environmental context. Thus, our research demonstrates that is not enough to provide the opportunities or encouragement to craft for employees to start exhibiting these behaviors. Our line of work suggests that organizations may benefit from introducing elements enhancing job crafting self-efficacy during job crafting interventions to increase the probability that employees implement job crafting practices in their jobs. Moreover, the results of Study 2 suggest that JCSE, being a personal resource, is linked with enhanced work engagement and reduced job burnout. Therefore, our research suggests that there is an opportunity to affect the level of the aforementioned variables through JCSE psychological interventions.

The final contribution of our research is that the JCSES-9 may be helpful in monitoring employees' beliefs about their capability to modify demands and resources present at their job. This instrument may be used as one of the outcome evaluation tools of job crafting interventions.

### Limitations and future research

Our research has some limitations. In all of our studies we applied a cross-sectional (Study 1, Study 2, and Study 3) or time-lagged cross-sectional design (Study 4), which precludes causal inferences. Further studies using a longitudinal design need to be done to explore relationships between JCSE and related constructs. However, the main goal of our series of studies was to validate a scale to measure JCSE and a cross-sectional design is sufficient for these strictly psychometric purposes. Both the strength and directions of the obtained relationships support the theoretical validity of the JCSE Scale. Another limitation is that in all studies we used self-report measures, which may lead to common method variance (monomethod bias; [66]). To minimize this risk we avoided repeated use of the same anchor points in questionnaires, and in Study 4 we spaced the predictors and explained variables in time [67]. To further reduce this method bias, future studies could also include various types of measures, such as peer-ratings or supervisor assessments of job crafting behaviors [29].

Moreover, the results of our research suggest that JCSE in increasing social resources may operate differently than other JCSE dimensions. Results of the regression analysis showed that the impact of GSE on increasing social resources ceases to be significant when JCSE in increasing social resources enters the model. With regard to environmental factors—when the dimension of JCSE in increasing social resources enters the regression model as a predictor, the influence of perceived opportunity to craft in an organization on this parallel job crafting dimension starts to be negative. Future studies need to examine how JCSE in increasing social resources may operate in relationship with related constructs.

## Conclusion

Job crafting is said to occur in every profession [68], on every organizational level [69], and on an everyday basis [70]. In our research we introduced the concept of JCSE, which may constitute one of the key predictors of job crafting behaviors. We also developed and validated a measure to assess JCSE, which may be used by both researchers and practitioners to empirically examine this phenomenon.

## Supporting information

S1 Appendix Qualtitative Study—interviews about barriers to job crafting. (DOCX)

S2 Appendix Multigroup confirmatory factor analysis and invariance test. (DOCX)

S3 Appendix Final version of the JCSE scale. (DOCX)

S1 Table Means, standard deviations, factor loadings, Cronbach’s alphas of the JCSE scale-27 and its correlations with JCSE dimensions in Study 1 (N = 364). (DOCX)
