# Supplementary material for: I believe I can craft! introducing Job Crafting Self-Efficacy Scale (JCSES)
Source: PLoS One. 2020 Aug 10;15(8):e0237250. doi: 10.1371/journal.pone.0237250 (PMC7416938; doi:10.1371/journal.pone.0237250)
Supplement: S1 Appendix — (DOCX) [file pone.0237250.s001.docx]

**S1 Appendix**

**Qualtitative Study—interviews about barriers to job crafting**

The aim of the interviews was to better understand what barriers hinder job crafting behaviors and to use this knowledge to build self-efficacy items including these specific impediments, as the literature suggests (Schwarzer, & Fuchs, 1996). For this purpose we conducted 20 semi-structured interviews. The interviews were conducted by one person knowledgeable in job crafting and self-efficacy theories. The sample for the interviews was composed of Polish employees from various occupational contexts (e.g., a teacher, a soldier, or a sales person) working for distinct organizations (e.g., small local companies, international corporations, public organizations). The respondents were diversified in terms of such demographic characteristics as gender (10 woman, 10 man), age (28–53), education (basic to higher educated), tenure (3–30), as well as the rank of their position (lower-level position to director).

The qualitative study resulted in a list of common and specific barriers to job crafting behaviors. All participants mentioned obstacles at different levels, that is, personal (e.g., low self-esteem), team (e.g., hostile relations among employees), and organizational (e.g., lack of autonomy). Some barriers were common and typical for all types of crafting, for example, fear of being negatively judged by colleagues. At the same time, participants declared some specific obstacles to each proactive type of crafting: increasing structural resources (e.g., lack of competent team), increasing social resources (e.g., lack of trust among team members), increasing challenging demands (e.g., fear of being overwhelmed with additional tasks).
