# Supplementary material for: I believe I can craft! introducing Job Crafting Self-Efficacy Scale (JCSES)
Source: PLoS One. 2020 Aug 10;15(8):e0237250. doi: 10.1371/journal.pone.0237250 (PMC7416938; doi:10.1371/journal.pone.0237250)
Supplement: S2 Appendix — (DOCX) [file pone.0237250.s002.docx]

**S3 Appendix**

**Multigroup Confirmatory Factor Analysis and Invariance Test**

**Paper-and-pencil versus online versions**

In order to ensure that both formats (online and paper-and-pencil) are comparable, we tested measurement invariance by performing a series of nested model comparisons between a default model and more constrained models. In the default model, all factor loadings, all factor variances, and all factor covariances were freely estimated across format conditions (see Model 1, Table A). First, we compared the default model with the fully constrained model (see Model 2, Table A) in which all factor loadings, all factor variances, and all factor covariances were constrained to be equal in both the paper-and pencil and online samples. Compared to the default model, the fit of the fully constrained model deteriorated significantly, Δχ² = 26.39, Δ*df* = 12, *p*<.01, meaning that some equality constraints do not hold across the two format conditions. Then, to test metric equivalence, we compared the model with factor loadings constrained to be equal across both format conditions (see Model 3, Table A) with the default model. There was no significant difference between these two models, indicating that factor loadings are invariant across samples, Δχ² = 5.89, Δ*df* = 6, *p* = .44. Next, we compared the model with both factor loadings and factor variances constrained to be equal across format conditions (see Model 4, Table A) with the default model. Model 4 did not differ from the default model, Δχ² = 13.62, Δ*df* = 9, *p* =.14, suggesting that all factor loadings and all factor variances are equivalent across format conditions. Because, compared with the default model, the fully constrained model differed significantly in terms of fit indices, this result implies that covariances are not invariant across both format conditions. Constraining the covariances one by one and comparing such created models with the default model revealed all of the covariances to be noninvariant across paper-and pencil and online samples. The factor covariances are in the same direction in both samples, but in the paper-and pencil sample, they are significantly higher than in the online sample. Therefore, Model 4 may be accepted as the final model. The results of the invariance test suggest that all factor loadings and all factor variances are equivalent across the format conditions. Because all factor covariances are noninvariant, these findings support a moderate equivalence of the online and paper-and-pencil versions of the JCSE Scale (Byrne, 2004).

Table A

*Multigroup Confirmatory Factor Analysis and Invariance Test of the JCSES-9: Paper-and-Pencil versus Online Versions (N =* *364 and N =* *432)*

| Model | *χ^2^* | *df* | *χ^2^/df* | RMSEA [90% CI] | CFI | TLI |
| --- | --- | --- | --- | --- | --- | --- |
| One-factor model (9 items) | 348.14 | 54 | 6.45 | 0.08 [0.075–0.091] | .86 | .82 |
| Three-factor model (9 items) | 156.92 | 48 | 3.27 | 0.05 [0.044–0.063] | .95 | .92 |
| Model 1 (default model) | 156.92 | 48 | 3.27 | 0.05 [0.044–0.063] | .95 | .92 |
| Model 2 (fully constrained) | 183.31 | 60 | 3.05 | 0.05 [0.043–0.059] | .94 | .93 |
| Model 3 (factor loadings constrained) | 162.80 | 54 | 3.01 | 0.05 [0.042–0.059] | .95 | .93 |
| Model 4 (factor loadings and factor variances constrained) | 170.54 | 57 | 2.99 | 0.05 [0.042–0.059] | .95 | .93 |

*Note.* χ2 = chi-square; *df* = degrees of freedom; *χ²/df* = normed chi-square; RMSEA = Root Mean Square Error of Approximation; CFI = Comparative Fit Index; TLI = Tucker-Lewis Index.

**Polish versus US versions**

In order to verify whether the three-factor model was invariant across Polish and US samples we first tested the default model in which no equality constraints were imposed (see Model 1, Table 5). Compared with the default model, the fully constrained model (see Model 2, Table 5) differed significantly in terms of fit indices, Δχ² = 24.50, Δ*df* = 12, *p*<.05. Further analyses revealed that both the model with factor loadings (see Model 3, Table 5; Δχ² = 5.88, Δ*df* = 6, *p* = .44) and the model with factor loadings and factor variances constrained to be equal across both samples (see Model 4, Table 5; Δχ² = 8.13, Δ*df* = 9, *p*=.52) did not differ from the default model. This indicates that all factor loadings and all factor variances are equivalent across Polish and US samples. Because the fully constrained model differed significantly from the default model it also implies that factor covariances were not invariant across both samples. To find out which specific covariances were not equivalent, we constrained the covariances one by one and compared such created models with the default model. The results from this series of tests revealed only the covariance between JCSE in increasing structural job resources and JCSE in increasing social job resources to be noninvariant across samples. The aforementioned factor covariance is in the same direction in both samples, but in the US sample is significantly higher than in the Polish sample. The final model (see Model 5, Table 5) did not differ from the default model, Δχ² = 12.73, Δ*df* = 11, *p* = .31. In sum, all factor loadings, all factor variances and two of the three factor covariances were equivalent across the Polish and US samples. These findings support invariance of the JCSE Scale, indicating the robustness of the scale (Byrne, 2004).

Table B

*Multigroup Confirmatory Factor Analysis and Invariance Test of the JCSES-9: Polish versus US versions (N=432 and N=403)*

| Model | *χ^2^* | *df* | *χ^2^/df* | RMSEA [90% CI] | CFI | TLI |
| --- | --- | --- | --- | --- | --- | --- |
| One-factor model (9 items) | 372.56 | 54 | 6.90 | 0.08 [0.076–0.092] | .86 | .82 |
| Three-factor model (9 items) | 150.10 | 48 | 3.13 | 0.05 [0.042–0.060] | .96 | .93 |
| Model 1 (default model) | 150.10 | 48 | 3.13 | 0.05 [0.042–0.060] | .96 | .93 |
| Model 2 (fully constrained) | 174.60 | 60 | 2.91 | 0.05 [0.040–0.056] | .95 | .94 |
| Model 3 (factor loadings constrained) | 155.98 | 54 | 2.89 | 0.05 [0.039–0.056] | .96 | .94 |
| Model 4 (factor loadings and factor variances constrained) | 158.23 | 57 | 2.78 | 0.05 [0.038–0.055] | .96 | .94 |
| Model 5 (final model) | 162.83 | 59 | 2.76 | 0.05 [0.038–0.054] | .96 | .95 |

*Note.* χ2 = chi-square; *df* = degrees of freedom; *χ²/df* = normed chi-square; RMSEA = Root Mean Square Error of Approximation; CFI = Comparative Fit Index; TLI = Tucker-Lewis Index.
