# Supplementary material for: I believe I can craft! introducing Job Crafting Self-Efficacy Scale (JCSES)
Source: PLoS One. 2020 Aug 10;15(8):e0237250. doi: 10.1371/journal.pone.0237250 (PMC7416938; doi:10.1371/journal.pone.0237250)
Supplement: S3 Appendix — (DOCX) [file pone.0237250.s003.docx]

**S4 Appendix**

**Final version of the JCSE Scale**

On a scale from 1 – ‘definitely incapable’ to 10  - ‘definitely capable’ please relate the following statements to your job and rate how certain you are that you would be capable to:

1. Introduce improvements to the way you perform your job, despite a lack of energy.
2. Seek opportunities for development at work despite minimal support from co-workers and supervisor.
3. Ask your supervisor for feedback, even though he/she doesn’t give it on his/her own account.
4. Set new job challenges, even though it leads to more responsibilities or a larger workload.
5. Ask your supervisor for support, despite concerns about being judged.
6. Perform tasks that go beyond your job description, despite concerns of how this may affect your personal life.
7. Seek advice from your co-workers, despite their own high workload.
8. Create and initiate new projects, even though the work environment is not supportive.
9. Develop your knowledge and abilities, despite a heavy workload.
