# Supplementary material for: I believe I can craft! introducing Job Crafting Self-Efficacy Scale (JCSES)
Source: PLoS One. 2020 Aug 10;15(8):e0237250. doi: 10.1371/journal.pone.0237250 (PMC7416938; doi:10.1371/journal.pone.0237250)
Supplement: S1 Table — (DOCX) [file pone.0237250.s004.docx]

**S2 Table**

Means, standard deviations, factor loadings, Cronbach’s alphas of the JCSE Scale-27 and its correlations with JCSE dimensions in Study 1 (N = 364)

| JCSES Factor | Item | *M* | *SD* | Factor loading | Cronbach’s Alpha Reliability |
| --- | --- | --- | --- | --- | --- |
|  |  |  |  |  |  |
| Increasing structural resources | jcse1 | 6.23 | 2.22 | 0.68*** | .90 |
| (IStR) | jcse2 | 6.47 | 2.11 | 0.68*** |  |
|  | jcse5 | 6.55 | 2.22 | 0.79*** |  |
|  | jcse6 | 6.59 | 2.21 | 0.79*** |  |
|  | jcse12 | 6.75 | 2.16 | 0.80*** |  |
|  | jcse18 | 6.24 | 2.27 | 0.68*** |  |
|  | jcse20 | 6.48 | 2.23 | 0.58*** |  |
|  | jcse25 | 6.92 | 2.19 | 0.76*** |  |
| Increasing social resources | jcse3 | 7.00 | 2.31 | 0.58*** | .88 |
|  | jcse7 | 5.21 | 2.53 | 0.72*** |  |
| (ISoR) | jcse8 | 5.49 | 2.48 | 0.79*** |  |
|  | jcse9 | 4.68 | 2.70 | 0.70*** |  |
|  | jcse16 | 6.02 | 2.58 | 0.80*** |  |
|  | jcse21 | 6.39 | 2.23 | 0.64*** |  |
|  | jcse22 | 5.62 | 2.60 | 0.76*** |  |
| Increasing challenging demands  (ICD) | jcse4 | 6.31 | 2.30 | 0.76*** | .86 |
|  | jcse13 | 6.68 | 2.29 | 0.79*** |  |
|  | jcse15 | 5.63 | 2.41 | 0.65*** |  |
|  | jcse17 | 5.71 | 2.54 | 0.74*** |  |
|  | jcse19 | 5.68 | 2.54 | 0.67*** |  |
|  | jcse24 | 5.69 | 2.27 | 0.66*** |  |
| JCSE in decreasing hindrance demands | jcse10 | 4.84 | 2.47 | 0.71*** | .80 |
| (DHD) | jcse11 | 4.94 | 2.55 | 0.59*** |  |
|  | jcse14 | 5.97 | 2.21 | 0.61*** |  |
|  | jcse23 | 5.23 | 2.55 | 0.51*** |  |
|  | jcse26 | 5.30 | 2.33 | 0.71*** |  |
|  | jcse27 | 5.54 | 2.17 | 0.71*** |  |

*Note.* JCSE = Job crafting self-efficacy; JC = Job crafting; IStR = increasing structural resources; ISoR = increasing social resources; ICD = increasing challenging demands; DHD = decreasing hindering demands.

* *p <* .05. ** *p <* .01. *** *p <* .001.
